# Disparities in Dental Services Use Among Urban and Rural Adults in China’s Megacities

**DOI:** 10.1093/geroni/igab046.113

**Published:** 2021-12-17

**Authors:** Xiaomin Qu, Xiang Qi, Bei Wu

**Affiliations:** 1 East China University of Political Science and Law, East China University of Political Science and Law, Shanghai, China (People's Republic); 2 Rory Meyers College of nursing, New York, New York, United States; 3 New York University, New York, New York, United States

## Abstract

Using data from the ‘2019 New Era and Living Conditions in Megacities Survey’ that included 4,049 residents aged 18-65, we examined the urban-rural disparities in dental visits among adults living in China’s 10 megacities. All of China’s megacities are metropolitan regions that include urban, peri-urban and rural land, and all have rural populations within the city boundaries. The results show that 43.3% (n=595) rural and 23.8% urban (n=637) residents had never visited dentists. Urban residents were more likely to visit dentists than rural residents after controlling for covariates (OR=1.57, 95%CI=1.30 to 1.91). The rates of visits were similar across age groups. Higher socioeconomic status, having urban insurances, having positive attitudes towards healthy diets and visited physicians regularly, and having poorer oral health was associated with higher odds of visiting dentists (P<0.05). These findings can help develop policies to increase dental care access to underserved populations in Chinese megacities.

